# Complex Visual Hallucinations in Charles Bonnet Syndrome: Diagnostic and Treatment Challenges in a Case with Psychotic Features

**DOI:** 10.1192/j.eurpsy.2025.1752

**Published:** 2025-08-26

**Authors:** A. Romero Teruel, A. Vives Luengo, B. Franco Lovaco

**Affiliations:** 1 Psychiatry, Hospital Dr. Rodríguez Lafora, Madrid, Spain

## Abstract

**Introduction:**

A 64-year-old male with diabetes-related blindness was admitted with anxiety, low mood, and passive suicidal ideation. Despite no psychiatric history, he experienced visual hallucinations, which he recognized as “optical illusions.” A CT scan ruled out organic causes, and retinal detachment with irreversible vision loss was confirmed. Diagnosed with Charles Bonnet Syndrome (CBS), he developed delusional interpretations and auditory hallucinations. Initially treated with Venlafaxine 150 mg/day, his psychotic symptoms persisted until Risperidone was increased to 6 mg/day, which resolved the hallucinations. Lithium was then added, allowing a reduction in Risperidone without relapse.

**Objectives:**

This review, based on a case study, focuses on the challenges of treating Charles Bonnet Syndrome (CBS), early diagnosis, and the lack of clear diagnostic criteria. It explores the connection between CBS and visual loss, the emergence of psychotic symptoms, and the role of antipsychotics in managing them.

**Methods:**

Literature was reviewed using the following keywords: (Charles Bonnet syndrome) AND (visual hallucinations OR hallucinations) AND (treatment OR management OR therapy OR pharmacotherapy). Databases such as PubMed were used to gather relevant studies.

**Results:**

There are several challenges in diagnosing and treating CBS. Underdiagnosis is common, leading to misdiagnoses such psychosis (Voit et al., 2021; Stojanov, 2016). Non-pharmacological approaches, including improving vision aids, have shown effectiveness in reducing hallucinations (Yacoub & Ferrucci, 2011; Pang, 2016).However, pharmacological treatments, including antipsychotics and SSRIs, have shown inconsistent results (Voit et al., 2021; Rojas & Gurnani, 2023). Differentiating CBS from psychiatric disorders is crucial, as patients typically retain awareness of the unreal nature of their hallucinations (Stojanov, 2016). Emerging research on CBS neurobiology suggests potential for future targeted therapies (Weil & Lees, 2021; Collerton et al., 2023).

**Conclusions:**

Charles Bonnet Syndrome (CBS) is frequently underdiagnosed due to limited awareness and patient underreporting. Non-pharmacological approaches, such as improving vision and social support, help alleviate symptoms, though no standardized pharmacological treatments exist. This case underscores the importance of distinguishing CBS from psychiatric disorders, especially when psychotic features are present. A multidisciplinary approach, involving ophthalmologists, psychiatrists, and neurologists, is essential for effective management, as seen in this case. Early diagnosis and ongoing research are crucial for developing more targeted treatments for CBS.

**Disclosure of Interest:**

None Declared

